# Immune-related Plasma Proteins Associated with Recurrent Aphthous Stomatitis Identified by Mendelian Randomization and Clinical Validation

**DOI:** 10.3290/j.ohpd.c_2834

**Published:** 2026-10-21

**Authors:** Yongkang Wang, Jian Che, Cuiqiang Guan, Hongbo Guo

**Affiliations:** a Yongkang Wang Resident Physician, Department of Stomatology, Shanxi Bethune Hospital, Tongji Shanxi Hospital, Third Hospital of Shanxi Medical University, Shanxi Academy of Medical Sciences, Taiyuan, Shanxi Province, China. Conceptualization, methodology, investigation, data curation, formal analysis, prepared original draft, revised the manuscript.; b Jian Che Doctoral Candidate, Department of Oral Medicine, First Hospital of Shanxi Medical University, Taiyuan, Shanxi Province, China. Conceptualization, methodology, formal analysis, investigation, supervision, prepared original draft, reviewed and edited the manuscript, final approval of the manuscript.; c Cuiqiang Guan Chief Physician, Department of Oral Medicine, First Hospital of Shanxi Medical University, Taiyuan, Shanxi Province, China. Validation, supervision, clinical guidance, reviewed and edited the manuscript.; d Hongbo Guo Chief Physician, Department of Traditional Chinese Medicine, First Hospital of Shanxi Medical University, Taiyuan, Shanxi Province, China. Resources, supervision, clinical guidance, reviewed and edited the manuscript.

**Keywords:** immune-related plasma proteins, Mendelian Randomization, recurrent aphthous stomatitis, single-cell RNA sequencing.

## Abstract

**Purpose:**

To identify immune-related plasma proteins causally associated with susceptibility to recurrent aphthous stomatitis (RAS) through two-sample Mendelian randomization, and to validate their biological and clinical relevance using integrative multi-omics analyses and clinical samples.

**Materials and Methods:**

Two-sample Mendelian randomization was performed using protein quantitative trait locus (pQTL) data for immune-related plasma proteins from the deCODE genetics database and genome-wide association study (GWAS) summary statistics for RAS to identify proteins with potential causal associations. Candidate proteins were further evaluated by Gene Ontology (GO)/Kyoto Encyclopedia of Genes and Genomes (KEGG) enrichment, protein–protein interaction (PPI) network, summary-data-based Mendelian randomization (SMR), and colocalization analyses. Single-cell transcriptomic data from oral mucosa were integrated to characterize the cellular expression patterns of the corresponding genes. Finally, plasma levels of the candidate proteins were measured by enzyme-linked immunosorbent assay (ELISA) in 35 patients with RAS (sampled during both the ulcerative and healing phases) and 35 healthy controls to validate their clinical relevance.

**Results:**

A total of 28 immune-related plasma proteins were identified as having potential causal associations with RAS through two-sample Mendelian randomization. Functional enrichment analysis indicated that these proteins were mainly involved in immune and inflammatory pathways, including natural killer (NK) cell-mediated cytotoxicity, Th1/Th2 cell differentiation, and the IL-17 signaling pathway. SMR and colocalization analyses highlighted IL17A, MMP1, and IL2RA as key candidate genes, with IL17A showing the strongest genetic evidence. Single-cell transcriptomic analysis further revealed distinct cell-type-specific expression patterns of these genes in the oral mucosa. Clinical validation confirmed statistically significant alterations in the plasma levels of IL17A, MMP1, and IL2RA across different stages of RAS.

**Conclusions:**

This integrative study combining genetic analysis, single-cell transcriptomics, and clinical validation identified IL17A, MMP1, and IL2RA as key immune-related molecules associated with RAS. Among them, IL17A showed the strongest genetic evidence and may serve as a promising biomarker and potential therapeutic target for RAS.

Recurrent aphthous stomatitis (RAS) is one of the most common oral mucosal diseases, characterized by recurrent, painful superficial ulcers that substantially impair patients’ quality of life. Current management is primarily limited to symptomatic relief and promotion of ulcer healing, whereas effective strategies to prevent recurrence remain unavailable.^1^ Although the pathogenesis of RAS has not been fully elucidated, accumulating evidence suggests that it is a multifactorial immune-mediated disease in which genetic susceptibility, immune dysregulation, and environmental factors collectively contribute to disease development.^13^ Among these, aberrant T-cell-mediated immune responses and dysregulated inflammatory cytokine production, are considered to play a central role in the initiation and progression of RAS.^22^

The role of T-cells in RAS has traditionally been interpreted within the Th1/Th2 immune balance paradigm.^5^ The discovery of Th17 cells has expanded this concept, highlighting the contribution of the Th17/IL-17A axis to RAS pathogenesis. Increased circulating levels of Th17-related cytokines, including IL-17A, IL-6, and TNF-α, have been reported in patients with RAS, with IL-17A levels correlating with disease activity.^20^ Given that T-cell responses are mediated through cytokine networks, circulating immune mediators may serve as potential biomarkers reflecting systemic immune dysregulation and mucosal inflammation.

Emerging evidence indicates that circulating immune mediators not only reflect systemic immune status but may also participate in local immune regulation of the oral mucosa, thereby influencing the development and healing of RAS lesions.^20^ Consequently, immune-related plasma proteins have attracted increasing attention as potential biomarkers and therapeutic targets. However, conventional observational studies are susceptible to confounding and reverse causality, making it difficult to establish causal relationships between plasma immune proteins and RAS.^15^ Mendelian randomization (MR), which uses genetic variants as instrumental variables for causal inference, can effectively minimize these biases and has become a powerful approach for identifying causal biomarkers and therapeutic targets in complex diseases.^12^

Therefore, this study employed a two-sample Mendelian randomization approach to systematically identify immune-related plasma proteins potentially associated with RAS. Functional enrichment, SMR, colocalization analysis, single-cell transcriptomic analysis of oral mucosal tissues, and clinical validation were subsequently performed to comprehensively evaluate the genetic evidence, biological functions, and clinical relevance of the identified candidate proteins. Our findings are expected to provide new insights into the immunopathogenesis of RAS and facilitate the identification of potential biomarkers and therapeutic targets for the disease.

## MATERIALS AND METHODS 

### Ethical Approval

The clinical component of this study was approved by the Ethics Committee of First Hospital of Shanxi Medical University (approval number: KYLL-2025-038) and was conducted in accordance with the Declaration of Helsinki. All participants provided written informed consent. The deCODE pQTL, eQTLGen eQTL, RAS GWAS, and GSE164241 single-cell RNA sequencing datasets used in this study are publicly available and were accessed in accordance with their respective data-use policies. As this study was based exclusively on de-identified, publicly available data, additional ethical approval and informed consent were not required.

### Study Design

This study integrated genetic discovery and clinical validation to identify immune-related plasma proteins associated with RAS. Two-sample MR, SMR, and colocalization analyses were performed using plasma protein quantitative trait locus (pQTL) and RAS genome-wide association study (GWAS) summary statistics, followed by single-cell RNA sequencing analysis to characterize cellular expression patterns. Plasma levels of IL17A, MMP1, and IL2RA were then measured by enzyme-linked immunosorbent assay (ELISA) in 35 patients with RAS and 35 matched healthy controls to validate the genetic findings.

### Data Sources

Plasma protein pQTL summary statistics were obtained from deCODE genetics, including genetic association data for 4719 plasma proteins.^24^ Immune-related genes were retrieved from MSigDB using the GO:0006955 (immune response) gene set.^11^ GWAS summary statistics for RAS were obtained from the FinnGen consortium, with cases defined according to the ICD-10 code K12.03. eQTL summary statistics used for SMR analysis were obtained from the eQTLGen Consortium, comprising 31,684 peripheral whole-blood samples.^21^ Single-cell RNA-seq data were obtained from the GEO database (GSE164241) and included healthy human buccal mucosal tissue samples for cell type annotation and localization analysis of candidate protein expression.^23^

### Two-sample Mendelian randomization

Two-sample MR was performed using pQTL data as the exposure and RAS GWAS summary statistics as the outcome. Instrumental variables were selected at < 1 × 10^-6^, followed by linkage disequilibrium clumping (r^2^ < 0.001, 10,000 kb) and exclusion of weak instruments (F > 10). Causal effects were primarily estimated using the inverse-variance weighted (IVW) method, with MR-Egger, weighted median, weighted mode, and simple mode analyses performed as sensitivity analyses. Heterogeneity, horizontal pleiotropy, and multiple testing were assessed using Cochran’s Q test, the MR-Egger intercept, and false discovery rate (FDR) correction, respectively.^16^

### Functional Enrichment and Network Analysis

Genes encoding the candidate plasma proteins were subjected to GO and KEGG enrichment analyses using the clusterProfiler R package. PPI and pathway interaction networks were constructed using the STRING database and visualized in Cytoscape.^4^ Hub proteins were identified based on network topological parameters, including degree and betweenness centrality.

### Summary-data-based MR and Colocalization Analysis

SMR was performed using expression quantitative trait locus (eQTL) summary statistics and RAS GWAS data to prioritize candidate genes. The heterogeneity in dependent instruments (HEIDI) test was applied to distinguish pleiotropy from linkage disequilibrium, with P_HEIDI > 0.05 indicating a shared causal variant.^7^ Colocalization analysis was conducted using the coloc R package, and a posterior probability of colocalization (PPH4) ≥ 0.80 was considered strong evidence of a shared causal variant.^7^

### Cellular Localization in Oral Mucosal Single-cell Transcriptomes

The publicly available single-cell RNA sequencing dataset GSE164241 was re-analyzed to characterize the cellular expression of candidate genes in healthy human oral mucosa. Data processing, clustering, and cell-type annotation were performed using the Seurat R package. The expression of IL17A, MMP1, and IL2RA was visualized using uniform manifold approximation and projection (UMAP).^3^

### Clinical Validation Cohort

A prospective case–control study with paired within-patient comparisons was conducted. RAS was diagnosed according to previously reported clinical criteria based on recurrent episodes of painful oral ulceration, characteristic clinical features, and exclusion of other ulcerative disorders. Based on the estimated effect size, a minimum of 24 participants per group was required to achieve 80% statistical power, and 35 patients with RAS and 35 matched healthy controls were ultimately enrolled. Patients diagnosed with RAS at the Department of Oral Medicine, First Hospital of Shanxi Medical University between March and September 2025 were recruited. Demographic and clinical characteristics were collected using a standardized case report form, and RAS was classified into minor, major, and herpetiform subtypes. Peripheral blood samples were obtained from RAS patients during active and healing stages and from healthy controls at enrollment. Baseline characteristics are presented in Table 1.

**Table 1:** Baseline demographic and clinical characteristics of patients with recurrent aphthous stomatitis and healthy controls

Demographic characteristic	RAS group (n = 35)	Control group (n = 35)	p-value
Age in years, mean ± SD	29.6 ± 6.3	28.2 ± 5.9	0.418
BMI (kg/m^2^) mean ± SD	21.5 ± 2.3	22.2 ± 2.3	0.170
Sex, n (%)			0.630
Male	14 (40%)	17 (48.6%)	
Female	21 (60%)	18 (51.4%)	
Smoking (current/former), n (%)	11 (31.4%)	6 (17.1%)	0.012
Alcohol use, n (%)	8 (22.9%)	4 (11.4%)	0.028
Systemic diseases, n (%)	0 (0.0%)	0 (0.0%)	—
Concomitant medication, n (%)	0 (0.0%)	0 (0.0%)	—
Clinical characteristics (RAU group)			
Disease duration in years, median (IQR)	3.8 (2.25–5.05)	—	—
Annual recurrence episodes, median (IQR)	6 (5.0–8.5)	—	—
Pain VAS score (0–10), mean ± SD	6.02 ± 1.38	—	—
Ulcer diameter in mm, mean ± SD	4.45 ± 1.14	—	—
RAS subtype, n (%)			—
Minor (MiRAS)	31 (88.5%)	—	
Major (MjRAS)	4 (11.5%)	—	
Herpetiform (HU)	0 (0.0%)	—	


### Enzyme-linked Immunosorbent Assay (ELISA)

Plasma concentrations of IL17A, MMP1, and IL2RA were measured using commercially available ELISA kits (Proteintech, Wuhan, China, for IL17A and MMP1; R&D Systems, Minneapolis, MN, USA, for IL2RA) according to the manufacturers’ instructions. Absorbance was measured using a microplate reader, and protein concentrations were calculated from the corresponding standard curves.

### Statistical Analysis

Statistical analyses were performed using R software (version 4.4.2). Normality was assessed using the Shapiro–Wilk test. Because plasma IL17A, MMP1, and IL2RA levels were not normally distributed, log transformation was applied before analysis. Baseline characteristics were compared using the chi-squared test. Independent-samples t-tests were used to compare patients with healthy controls, and paired t-tests were used to compare the active and healing stages within patients. All tests were two-sided, with p < 0.05 considered statistically significant.

## RESULTS

The datasets used in the discovery stage of this study are publicly available. pQTL, GWAS, eQTL, and single-cell RNA sequencing data were obtained from deCODE genetics, the FinnGen consortium, the eQTLGen Consortium, and the Gene Expression Omnibus (GSE164241), respectively. Immune-related genes were retrieved from the Molecular Signatures Database (MSigDB; GO:0006955). The clinical validation data generated during this study are available from the corresponding author upon reasonable request.

### Screening for RAS-associated Immune-related Plasma Proteins

In this study, pQTL data for 4719 plasma proteins from the deCODE database were intersected with immune-related genes, yielding 577 immune-related proteins for two-sample Mendelian randomisation (MR) analysis. After false discovery rate (FDR) correction, 28 plasma proteins were identified as causally associated with recurrent aphthous stomatitis (RAS). Based on NCBI Gene annotations and published evidence, these proteins were classified into four functional categories: inflammatory response (10 proteins), antigen presentation (4 proteins), cell activation (7 proteins), and signal transduction (7 proteins). Overall, inflammatory response proteins were predominantly associated with increased RAS risk, whereas most proteins involved in antigen presentation, cell activation, and signal transduction showed protective effects. CRP exhibited the strongest protective effect (OR = 0.56, 95% CI: 0.39–0.80, p=0.001), whereas IL2RA (OR = 1.37, 95% CI: 1.01–1.86, p=0.045) and SMPD1 (OR = 1.20, 95% CI: 1.03–1.40, p=0.020) were among the strongest risk-associated proteins. The results are presented as forest plots (Fig 1).

**Fig 1:**
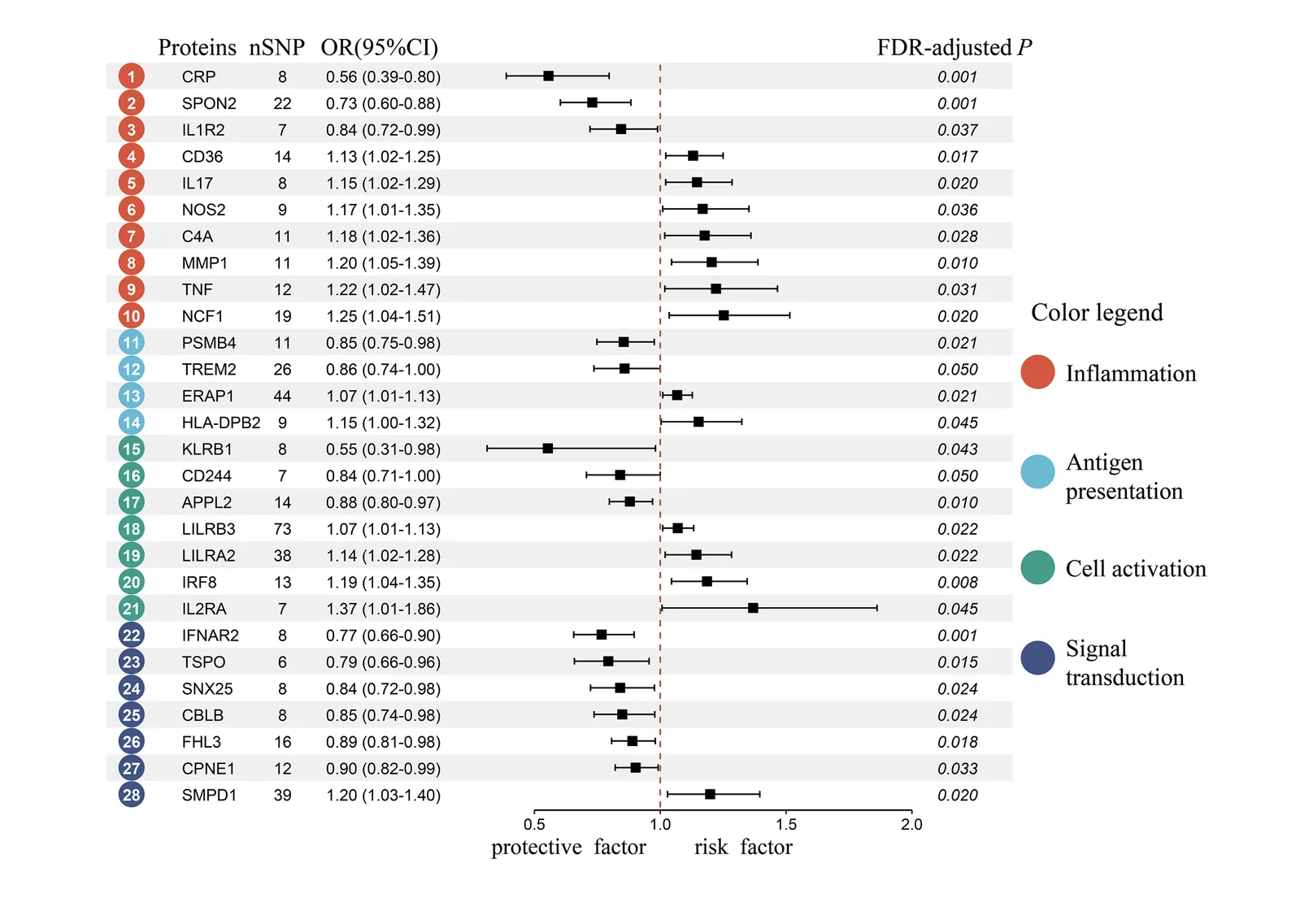
Forest plot of Mendelian randomization analysis between immune-related plasma proteins and RAS.

### Pathway Enrichment and Molecular Interaction Network Analysis

After identifying 28 immune-related plasma proteins associated with RAS, GO and KEGG enrichment analyses were performed to explore their biological functions and signaling pathways. GO analysis (Fig 2a) indicated that these genes were mainly enriched in biological processes related to responses to biotic stimuli, phagocytosis, and inflammatory mediator production; cellular components including membrane microdomains, membrane rafts, and phagocytic vesicles; and molecular functions associated with immune receptor activity and protein–lipid complex binding. KEGG analysis (Fig 2b) showed statistically significant enrichment in pathways related to natural killer cell-mediated cytotoxicity, Th1/Th2 cell differentiation, the IL-17 signaling pathway, and lipid and atherosclerosis, suggesting the involvement of immune regulation and inflammatory responses in RAS.

**Fig 2:**
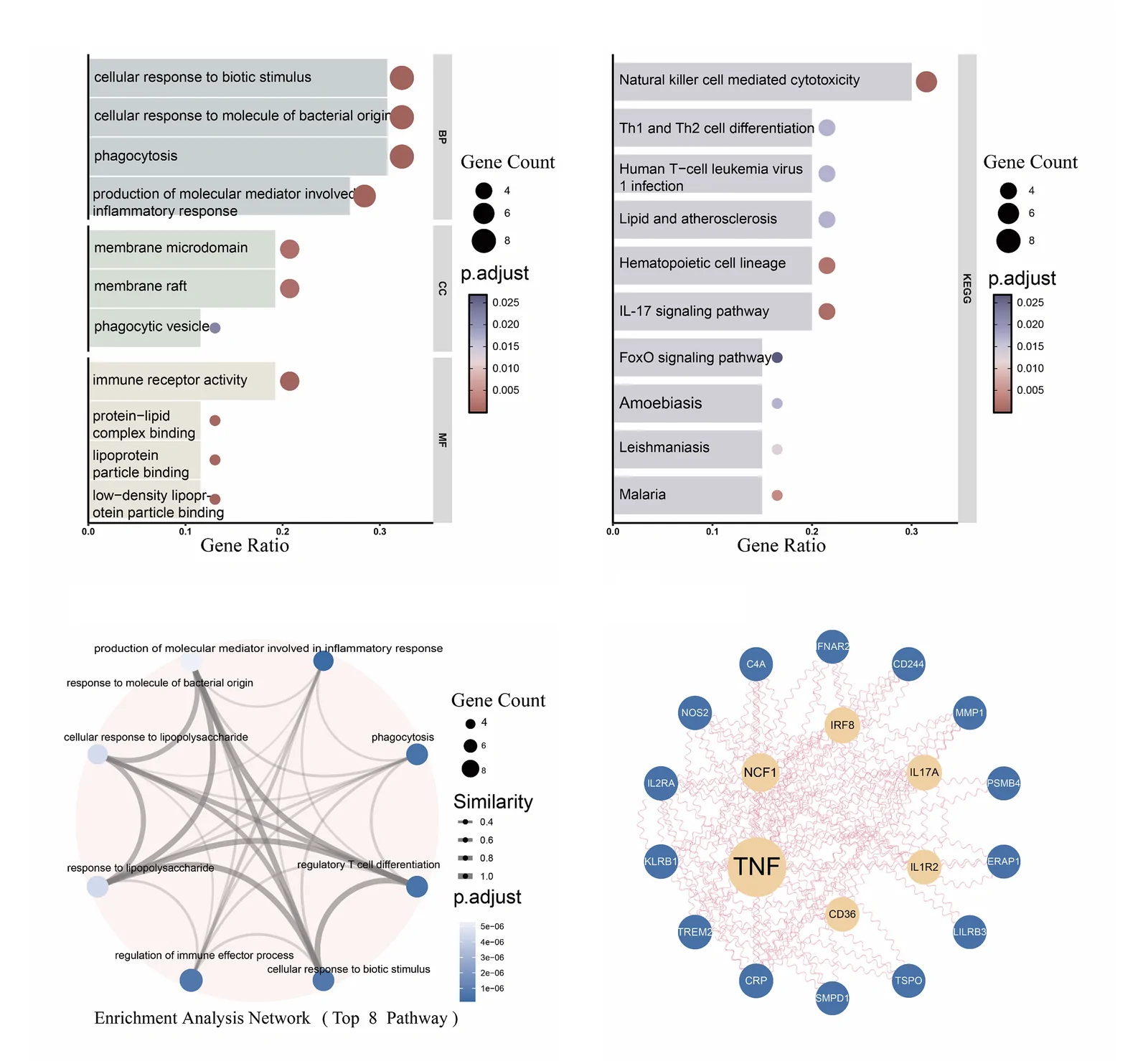
Functional enrichment and protein–protein interaction network of immune-related plasma proteins. (a) Bubble bar plot of GO functional enrichment analysis; (b) bubble bar plot of KEGG pathway enrichment analysis; (c) pathway similarity interaction network; (d) protein–protein interaction (PPI) network.

A pathway similarity network constructed from the top eight GO terms (Fig 2c) revealed extensive functional overlap among enriched pathways, indicating coordinated regulation of multiple immune-related biological processes. The protein–protein interaction network (Fig 2d) showed that most candidate proteins were highly interconnected, with TNF occupying a central position. Several proteins, including NCF1, IRF8, IL17A, IL1R2, and CD36, also exhibited strong interactions, suggesting that multiple immune-related proteins may cooperatively contribute to RAS pathogenesis.

### SMR and Colocalization Analyses

The eQTL data used for summary-data-based Mendelian randomization (SMR) analysis were obtained from the eQTLGen Consortium. Based on the 28 candidate proteins identified by MR, SMR analysis was performed to evaluate the association between gene expression and RAS risk. Three genes, IL17A, MMP1, and IL2RA, showed statistically significant associations with RAS (Table 2). HEIDI tests were statistically non-significant for all three genes (p>0.05), indicating no evidence of heterogeneity or horizontal pleiotropy.

**Table 2:** Results of the SMR and colocalization analyses of immune-related plasma proteins associated with recurrent aphthous stomatitis

Plasma protein	SMR Analysis				Colocalization analysis	
	b (SMR)	SE (SMR)	P(SMR)	P (HEIDI)	PPH3	PPH4
IL17A	0.54	0.08	0.001	0.412	0.03	0.95
MMP1	0.38	0.07	0.001	0.156	0.88	0.06
IL2RA	0.45	0.09	0.003	0.089	0.79	0.12


Colocalization analysis was subsequently performed to determine whether the eQTL and GWAS signals shared the same causal variant. IL17A showed strong evidence of colocalization (PPH4 = 0.95; PPH3 = 0.03), with substantial overlap between the eQTL and GWAS association signals and a shared lead variant (rs2275913) (Fig 3). In contrast, MMP1 and IL2RA showed low PPH4 values (0.06 and 0.12, respectively) and limited overlap between eQTL and GWAS signals, indicating no convincing evidence of colocalization.

**Fig 3:**
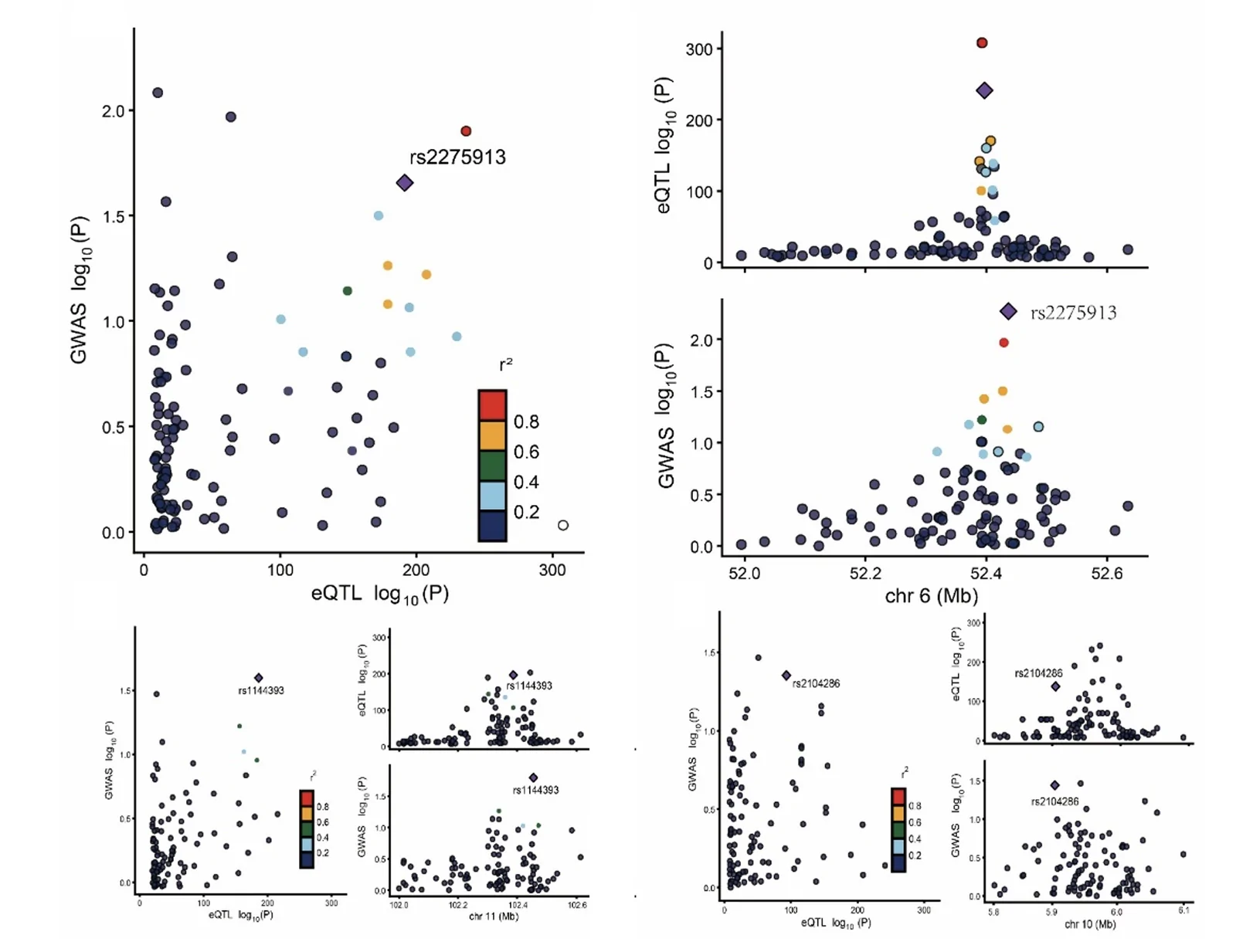
eQTL and GWAS signal colocalization plot. (a) IL17A; (b) MMP1; (c) IL2RA.

### Single-cell Analysis of Oral Mucosa

Based on the GSE164241 dataset, a single-cell transcriptomic atlas of healthy oral buccal mucosa was constructed. After quality control and dimensionality reduction, approximately 60,000 cells were retained and classified into eight major cell populations according to canonical marker genes, including epithelial cells, fibroblasts, endothelial cells, CD4⁺ T cells, CD8⁺ T/NK cells, myeloid cells, and mast cells (Fig 4a). UMAP visualization showed clear separation of these cell populations, supporting the reliability of the cell-type annotation (Fig 4b).

**Fig 4:**
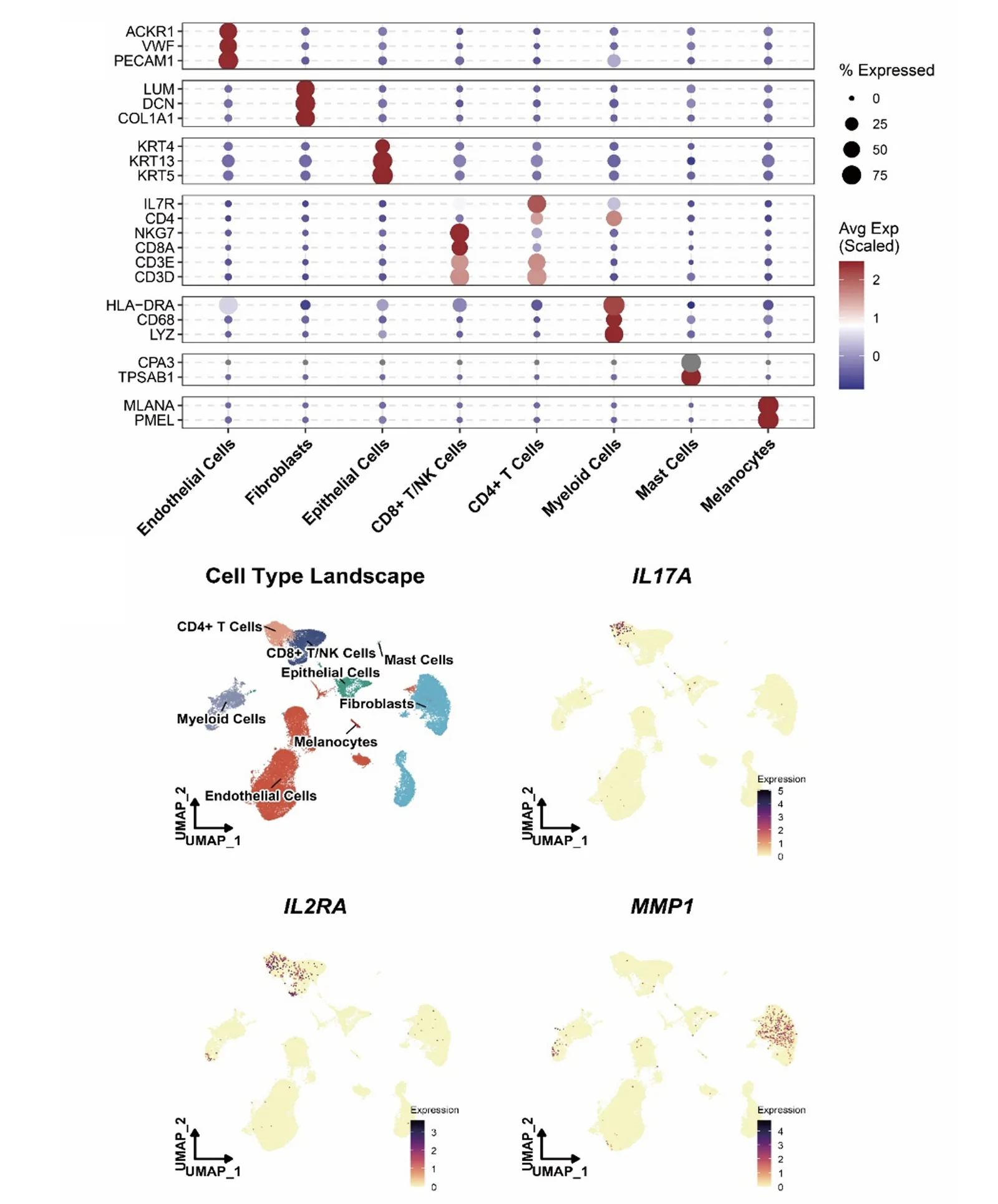
Cell-type annotation and target gene expression distribution in the single-cell transcriptome of oral mucosa. (a) Cell-type annotation of the oral mucosal single-cell transcriptomic data based on canonical epithelial marker genes; (b) UMAP visualization showing the distribution of distinct cell populations and the expression patterns of IL17A, IL2RA, and MMP1 across different cell subpopulations.

The expression patterns of IL17A, MMP1, and IL2RA were further examined at the single-cell level (Fig 4b). IL17A showed minimal expression in healthy oral mucosa. MMP1 was predominantly expressed in fibroblasts, indicating strong cell-type specificity. IL2RA was expressed at low levels and was mainly restricted to CD4⁺ T cells, with limited expression in CD8⁺ T/NK cells.

Clinical validation of dynamic changes in plasma proteins: ELISA results (Fig 5, Table 3) showed that IL17A, MMP1, and IL2RA exhibited different dynamic changes at different stages of RAS. IL17A was statistically significantly elevated during the ulcerative phase (FC = 2.36, p<0.001) and decreased during healing, although remaining slightly higher than controls (FC = 1.22, p=0.026), suggesting its potential role as an indicator of active inflammation. IL2RA showed a similar pattern, with increased levels during the ulcerative phase (FC = 1.79, p<0.001) and normalization after healing (p=0.665), indicating its association with disease activity. Although MMP1 did not differ statistically significantly during the ulcerative phase (FC = 1.27, p=0.483), its reduction during healing suggests a possible involvement in tissue repair. These dynamic alterations highlight the potential value of IL17A and IL2RA as biomarkers for monitoring RAS activity and recovery.

**Fig 5:**
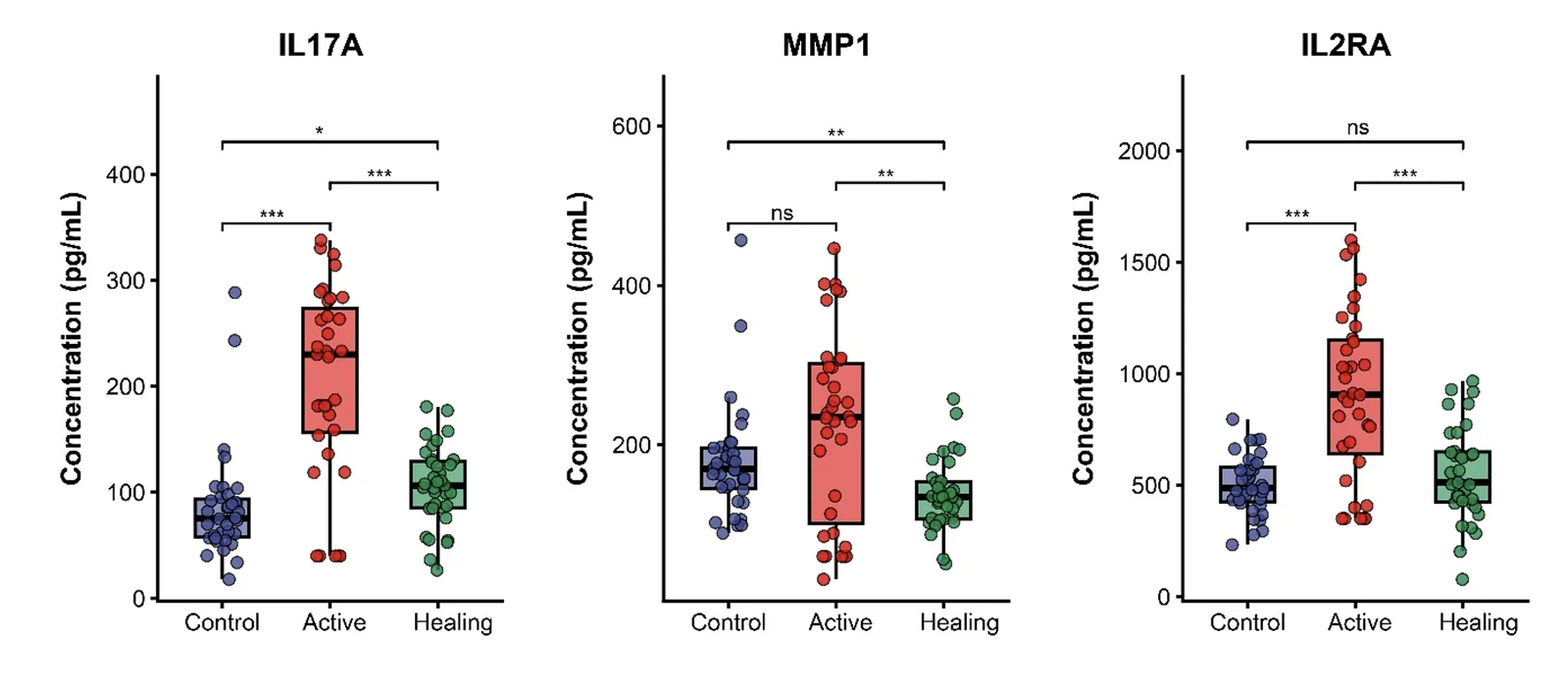
Plasma concentrations of IL17A, MMP1, and IL2RA in healthy controls and patients with recurrent aphthous stomatitis at different clinical stages.

**Table 3:** Comparison of plasma protein levels between healthy controls and patients with recurrent aphthous stomatitis at different clinical stages

Group/comparison	IL17A (pg/ml)	MMP1 (pg/ml)	IL2RA (pg/ml)
Healthy controls	75.8 (57.9,93.5)	169.4 (145.2, 195.6)	486 (426, 581)
Active RAS	230.0 (156.2, 273.2)	235.2 (101.2, 302.0)	906 (640, 1150)
Healing RAS	106.6 (85.1, 129.5)	134.5 (107.0, 153.5)	512 (424, 650)
FC (active vs control)	2.36	1.27	1.79
FC (healing vs control)	1.22	0.77	1.10
p (control vs active)	< 0.001 ***	0.483	< 0.001 ***
p (control vs healing)	0.026 *	0.003 **	0.665
p (active vs healing)	< 0.001 ***	0.010 **	< 0.001 ***


## DISCUSSION

To date, the exact etiology and pathogenesis of RAS remain unclear. Although Though RAS manifests as recurrent localized oral ulcers, increasing evidence suggests that its development is closely associated with systemic immune dysregulation and inflammatory responses. Because direct collection of ulcer tissue is limited by ethical and practical considerations, previous studies have mainly investigated inflammation-related proteins in plasma or saliva to explore disease mechanisms and identify potential biomarkers.^2^ In recent years, studies on plasma proteins associated with RAS have primarily focused on inflammation and immune regulation, acute-phase responses, and oxidative stress.^25^ Previous studies have demonstrated abnormal expression of pro- and anti-inflammatory cytokines, acute-phase proteins, and oxidative stress-related biomarkers in patients with RAS, suggesting that immune dysregulation, systemic inflammation, and oxidative stress collectively contribute to the pathogenesis of RAS.^6^ Although circulating biomarkers cannot fully represent the local inflammatory microenvironment of oral ulcers, they may provide complementary information regarding systemic immune dysregulation associated with RAS and its local mucosal inflammatory responses.^10^

In the present study, we integrated pQTL data with immune-related genes and identified 28 immune-related plasma proteins with potential causal associations with RAS using two-sample MR and FDR correction, providing reliable candidate targets for subsequent validation. Functional annotation showed that these proteins were mainly involved in immune regulation, inflammatory responses, antigen presentation, immune cell activation, and signal transduction, further highlighting the important role of immune dysregulation in RAS.^18^

SMR integrates xQTL summary statistics to evaluate genetically supported associations between molecular traits and disease, while the HEIDI test helps distinguish shared causal variants from linkage disequilibrium.^9^ To strengthen the genetic evidence, we further performed SMR and colocalization analyses. IL17A, MMP1, and IL2RA showed statistically significant positive associations with RAS risk, with IL17A demonstrating the strongest genetic evidence. The combined use of SMR and colocalization analyses provides more robust genetic evidence for candidate signals, improving the reliability of causal inference and prioritizing candidate targets for subsequent clinical validation.^17^ Colocalization analysis suggested that IL17A is likely to share a causal variant with RAS, whereas the associations of MMP1 and IL2RA may be attributable to linkage disequilibrium, further supporting IL17A as the strongest candidate target.

Previous scRNA-seq studies have established a cellular atlas of normal human oral mucosa, providing important insights into its cellular composition and immune homeostasis.^23^ To explore the cellular distribution of candidate genes, we analyzed publicly available scRNA-seq data from normal oral mucosa. Although no RAS-specific scRNA-seq datasets are currently available, this analysis provides a preliminary reference for the cellular localization of candidate proteins. Future studies incorporating scRNA-seq of RAS lesions are needed to further clarify disease-specific cellular and molecular mechanisms.

Clinical validation further supported the genetic findings. Plasma IL17A levels remained statistically significantly elevated during both the ulcerative and healing phases, suggesting its persistent involvement in inflammation and tissue repair. Although inflammatory cytokine dysregulation has been reported in RAS, circulating IL-17A findings remain inconsistent.^20^ Shen et al^19^ found statistically significantly increased IL-2, IL-6, TNF-α, and IFN-γ levels in 127 RAS patients compared with 20 controls, whereas IL-17A showed no statistically significant difference. Differences in disease stage, patient characteristics, or detection methods may account for this discrepancy. MMP1 was elevated during the ulcerative phase and decreased during healing, consistent with its role in ECM remodeling, while IL2RA was increased only during active disease, reflecting transient T-cell activation. Given the limited evidence regarding IL2RA in RAS, findings from related immune-mediated ulcerative disorders may provide supportive insights. For example, studies of Behçet’s disease, a disorder characterized by recurrent oral ulceration, have reported elevated serum IL2RA levels in active patients, suggesting that IL-2 receptor signaling may contribute to inflammatory ulcerative processes.^15^ Collectively, these findings support IL17A, MMP1, and IL2RA as potential biomarkers and candidate therapeutic targets involved in RAS pathogenesis.

Several limitations should be acknowledged. First, although MR minimizes confounding and reverse causality, potential horizontal pleiotropy cannot be completely excluded, and the available RAS GWAS dataset remains relatively small. Second, the present study established genetic associations but lacked mechanistic validation using cellular and animal models. Third, circulating protein levels may not fully reflect changes within the local oral microenvironment. Future studies integrating functional experiments with disease-specific scRNA-seq analyses will help clarify the biological roles of these candidate proteins and their contributions to RAS pathogenesis.

## CONCLUSION

This MR-based study identified 28 immune-related plasma proteins potentially associated with RAS. Integrated analyses, including SMR, colocalization, scRNA-seq, and clinical validation, highlighted IL17A, MMP1, and IL2RA as key candidate proteins involved in RAS pathogenesis. Among them, IL17A showed the strongest genetic evidence and was statistically significantly elevated during the active phase of RAS, suggesting its potential role as a biomarker and therapeutic target. Importantly, these immune signatures may improve RAS risk stratification and prevention. IL17A, supported by the strongest genetic evidence, may serve as both a potential therapeutic target and biomarker for predicting disease activity or recurrence. Further longitudinal studies are needed to validate these findings and explore whether targeting these immune pathways can reduce RAS recurrence.

## ACKNOWLEDGEMENT

This study was supported by the General Program of the Natural Science Foundation of Shanxi Province (Grant No. 20243021221303) and the Scientific Research Project of the Shanxi Administration of Traditional Chinese Medicine (Grant No. 2023ZYYA023).

## AI Statement

AI was used solely for language editing, including grammar correction, sentence restructuring, and improvement of English clarity and readability. All AI-assisted text was reviewed and revised by the corresponding authors, who take full responsibility for the manuscript.
